# Bacterial, fungal, and interkingdom microbiome features of exclusively breastfeeding dyads are associated with infant age, antibiotic exposure, and birth mode

**DOI:** 10.3389/fmicb.2022.1050574

**Published:** 2022-11-17

**Authors:** Timothy Heisel, Abigail J. Johnson, Sara Gonia, Abrielle Dillon, Emily Skalla, Jacob Haapala, Katherine M. Jacobs, Emily Nagel, Stephanie Pierce, David Fields, Ellen Demerath, Dan Knights, Cheryl A. Gale

**Affiliations:** ^1^Department of Pediatrics, University of Minnesota, Minneapolis, MN, United States; ^2^School of Public Health, University of Minnesota, Minneapolis, MN, United States; ^3^HealthPartners Institute, Minneapolis, MN, United States; ^4^Department of Obstetrics, Gynecology, and Women’s Health, University of Minnesota, Minneapolis, MN, United States; ^5^College of Medicine, University of Oklahoma, Oklahoma City, OK, United States; ^6^Department of Computer Science and Engineering, University of Minnesota, Minneapolis, MN, United States

**Keywords:** bacteria, breastmilk, fungi, infant gut, microbiome, mycobiome, interkingdom interactions, microbial community variation

## Abstract

The composition and function of early life gut bacterial communities (microbiomes) have been proposed to modulate health for the long term. In addition to bacteria, fungi (mycobiomes) also colonize the early life gut and have been implicated in health disorders such as asthma and obesity. Despite the potential importance of mycobiomes in health, there has been a lack of study regarding fungi and their interkingdom interactions with bacteria during infancy. The goal of this study was to obtain a more complete understanding of microbial communities thought to be relevant for the early life programming of health. Breastmilk and infant feces were obtained from a unique cohort of healthy, exclusively breastfeeding dyads recruited as part of the Mothers and Infants Linked for Healthy Growth (MILk) study with microbial taxa characterized using amplicon-based sequencing approaches. Bacterial and fungal communities in breastmilk were both distinct from those of infant feces, consistent with niche-specific microbial community development. Nevertheless, overlap was observed among sample types (breastmilk, 1-month feces, 6-month feces) with respect to the taxa that were the most prevalent and abundant. Self-reported antibacterial antibiotic exposure was associated with micro- as well as mycobiome variation, which depended upon the subject receiving antibiotics (mother or infant), timing of exposure (prenatal, peri- or postpartum), and sample type. In addition, birth mode was associated with bacterial and fungal community variation in infant feces, but not breastmilk. Correlations between bacterial and fungal taxa abundances were identified in all sample types. For infant feces, congruency between bacterial and fungal communities was higher for older infants, consistent with the idea of co-maturation of bacterial and fungal gut communities. Interkingdom connectedness also tended to be higher in older infants. Additionally, higher interkingdom connectedness was associated with Cesarean section birth and with antibiotic exposure for microbial communities of both breastmilk and infant feces. Overall, these results implicate infant age, birth mode, and antibiotic exposure in bacterial, fungal and interkingdom relationship variation in early-life-relevant microbiomes, expanding the current literature beyond bacteria.

## Introduction

Infancy is a critical period of life when gut microbial communities (microbiomes) are maturing simultaneously and in interaction with all other infant physiological systems, including metabolism, immunity, and the brain. Indeed, the findings from many human and animal studies implicate microbial dysbiosis during infancy in the development of a wide range of disordered physiology (asthma and atopy, obesity, and mental health disorders; Ajslev et al., 2011; Diaz Heijtz et al., 2011; Huh et al., 2012; Arrieta et al., 2015; Murphy et al., 2015). Our understanding of how early life microbes are involved in later life health is almost entirely based on the study of bacteria. From this work, we know that the major drivers of early life bacterial microbiome variation are diet (breastmilk/formula), antibiotic exposure, and birth mode (Pannaraj et al., 2017; Stewart et al., 2018; Fehr et al., 2020). Breastmilk contains its own microbial communities and serves as an early microbial input to the developing infant gut. A recent study reported that during the first month of life, infants receive ~30% of their gut bacteria from breastmilk (Pannaraj et al., 2017).

Fungi (mycobiomes) are also present in breastmilk (Boix-Amorós et al., 2017; Heisel et al., 2019; Moossavi et al., 2020) and in the infant gut (Schei et al., 2017; Ward et al., 2017). Although fungi are thought to comprise only ~0.1% of microbial cells in the intestine, this likely underestimates their clinical relevance. Fungal cells are more than 100-fold larger than typical bacterial cells and thus provide substantial biomass to the microbiota as well as surface area for host-microbe and microbe-microbe interactions. In addition, fungi are capable of substantial proliferation under certain conditions (e.g., antibiotic pressure). Recent studies have shown associations between gut fungi and healthy, as well as disordered, immune development (Arrieta et al., 2015; Shao et al., 2019a; Mok et al., 2021).

Fungi and bacteria coexist and interact physically in most natural environments, communicate *via* molecules and induction of signaling pathways, and compete with or complement each other with respect to nutrient acquisition, metabolism, and growth (Peleg et al., 2010; Frey-Klett et al., 2011). Because of interkingdom interactions, mixed microbial communities often have functions that are significantly different from those of their component bacterial and fungal kingdom communities alone (Wargo and Hogan, 2006; Shirtliff et al., 2009) and medical therapies targeting one microbial kingdom have the potential to have effects on the other. For example, antibacterial antibiotic exposure is associated with variation in fungal community diversity in the gut of mice (Nettles et al., 2020). Thus, to gain a more complete understanding of how early life microbial communities contribute to health outcomes, knowledge about microbiome-mycobiome interactions is needed.

In this study, we characterize fungal and bacterial communities from a cohort of mothers and infants engaged in exclusive breastfeeding, thus eliminating potential dietary confounders that could contribute to microbial community variation. Our objectives were to discover interkingdom microbial relationships and to identify clinical factors associated with variation in microbiomes, mycobiomes, and their interkingdom relationships, both within human milk and the feces of the nursing infant.

## Materials and methods

### Subject enrollment and inclusion criteria

Healthy pregnant women, committed to exclusive breastfeeding for at least the first 3 months postpartum, were enrolled after informed consent as part of the prospective Mothers and Infants LinKed for Healthy Growth (MILk) study, a multi-site study in Oklahoma City, Oklahoma and the Minneapolis/St. Paul metropolitan area in Minnesota. The study is a collaboration between the University of Oklahoma Health Sciences Center, the University of Minnesota, and the HealthPartners Institute in Minneapolis, Minnesota. The Institutional Review Boards of the University of Oklahoma, the University of Minnesota, and the HealthPartners Institute approved this study. This study has been registered with^1^ (identifier NCT03301753). Study participants received stipends for completion of each sample collection per time point. The participants in the present study included a subset of MILk Study dyads (all from the Minnesota site) who consented to additional microbiome and mycobiome assessment of their milk and infant fecal samples.

Participants were included if they were non-smokers, non-diabetic, English speaking and understanding, and had delivered singleton infants at term gestation (37 0/7–42 1/7 weeks gestation) that were appropriately grown for gestational age (between the 10th and 90th percentile on WHO growth charts). Milk was obtained only if the dyad had been exclusively breastfeeding from birth to the time of the 1-month post-partum study visit. For this study of the microbiome and mycobiome, only samples from dyads that were engaged in exclusive breastfeeding at both the 1-month and the 6-month postpartum visits were included. Breastfeeding status was obtained by detailed self-report. Infants who received <24 oz. (720 ml) of formula since birth or their last study visit and only human milk for the 2 weeks prior to the study visit were considered exclusively breastfed. None of the women reported symptoms of mastitis or breast infection at the time of breastmilk collection.

### Clinical and demographic variables and collection methods

Clinical data for each breastfeeding dyad was collected from electronic medical records from the birth hospitalization and from questionnaires administered electronically at study visits at 1 and 6 months of infant age. Study data were collected and managed using REDCap electronic data capture tools hosted at the University of Minnesota. REDCap (Research Electronic Data Capture) is a secure, web-based software platform designed to support data capture for research studies (Harris et al., 2009, 2019).

Because not every dyad provided all sample types, we compared clinical characteristics among sample types to identify clinical feature differences that could potentially contribute to any observed microbiome differences by sample type (Supplementary Table S1). Clinical characteristics were also compared by sample type for the sub-group of samples used in the network analyses (Supplementary Table S2). The interkingdom network sub-group included all samples for which there were both bacterial and fungal sequence data available. Maternal pre-pregnancy BMI was estimated using the first available weight and height in the electronic medical record within the first 6 weeks of pregnancy. Antibiotic exposure was categorized by type (antibacterial or antifungal), timing (prenatal, perinatal (i.e., near the time of birth), during the first month postpartum, or from 1 month to 6 months postpartum), and the subject administered to (mother or infant). Antibiotic exposure information is compared by sample type (milk, 1-month fecal), 6-month fecal) for the overall cohort and network analysis sub-group in Supplementary Tables S3 and S4, respectively. Clinical characteristics are compared by delivery mode in Supplementary Table S5. Reporting of race in this study cohort was mandated by the US National Institutes of Health, consistent with the Inclusion of Women, Minorities, and Children policy. Maternal race was self-reported, and infant race was reported by the mother, *via* electronic survey that included six race categories (American Indian or Alaska native, Asian, Black or African American, More than one race, Other, White) and two ethnicity categories (Hispanic, non-Hispanic). For statistical analyses, non-white subjects were grouped together for comparison to white subjects, due to low numbers of non-white subjects. The following individual-level economic and dietary information were also collected *via* self-report: maternal education level, household income, and the NCI DHQ II food frequency questionnaire obtained at 1 month postpartum which was used to calculate the 2015 Healthy Eating Index (HEI) diet quality score (Krebs-Smith et al., 2018). Study questionnaires did not ask about parent gender identity and readers of this paper should be encouraged to read/use the gender-associated terms in this paper according to those with which they most identify, per the guidance of the Academy of Breastfeeding Medicine.

### Sample collection and storage

Breastmilk was obtained when infants were 1 month old along with a fecal sample; an additional fecal sample was obtained at 6 months of age. Breastmilk was collected using a hospital-grade electric breast pump (Medela Symphony; Medela, Inc., Zug, Switzerland), with complete expression of milk from the breast (i.e., continued until milk stopped flowing) to account for variations in milk concentration as previously described (Geraghty et al., 2005; Powe et al., 2010). The volume and weight of milk was recorded, milk was gently mixed, aliquots were made, and then stored at −80°C within 20 min of collection.

Feces produced during a study visit were collected from diapers aerobically using a sterile swab, placed into 2 ml cryovials, and stored within 30 min at −80°C. If a fecal sample was not produced during the study visit, subjects were provided with an at-home collection kit. For at-home sampling, feces were collected from diapers aerobically using a sterile swab and transferred to 2 ml cryovials containing 600 μl RNALater (Ambion/Invitrogen, Carlsbad, CA), placed into an envelope, and mailed to the University of Minnesota. Once received by the laboratory, fecal samples were stored at −20°C until use for DNA extraction. Near-immediate storage at −80°C (study visit collection) provides similar fecal microbiome feature extraction as storage in RNALater (home collection; Vogtmann et al., 2017).

### DNA extraction

For feces, cryovials containing thawed fecal suspensions were centrifuged at 13,000 RPM, the supernatant was removed, and fecal material was transferred to a fresh microfuge tube. DNA was extracted using the PowerSoil kit following the manufacturer’s instructions (QIAGEN, Germantown, MD), eluted with 100 μl of the provided elution solution, and stored in microfuge tubes at −80°C.

For DNA extraction from breastmilk, preliminary experiments were performed to evaluate DNA isolation methods with respect to quantity of microbial DNA recovered (Supplementary Table S6). Three DNA isolation kits were compared (PowerSoil, PowerFood, and PowerSoil Pro (all available from QIAGEN)) by qPCR using fungal (UNI1 and UNI2 (Heisel et al., 2015)) and bacterial-specific (515f and 806r (Caporaso et al., 2012)) primers. DNA yield was greatest when using the following process: Breastmilk (1 ml) was transferred to a sterile microfuge tube and centrifuged at 13,000 RPM. The supernatant and fat layer were removed, the pellet was resuspended in Solution 1 from the PowerSoil Pro kit, and this solution was then transferred to the first tube in the PowerSoil Pro kit. DNA extraction then proceeded following the manufacturer’s protocol. DNA was eluted with 100 μl of the provided elution solution and was stored in microfuge tubes at −80°C.

### Bacterial and fungal DNA sequencing

16S (bacterial) and ITS2 (fungal) DNA amplicons were generated from extracted DNA and sequenced by the University of Minnesota Genomics Center (UMGC, Minneapolis, MN). 16S amplicons were generated by a previously published method optimized by the UMGC (Gohl et al., 2016) using a dual-indexed PCR to amplify the V4 region of the bacterial rDNA locus using KAPA HiFi polymerase. ITS2 amplicons were generated using the same dual-index technique with a fungal primer set targeting the ITS2 region of the fungal rDNA locus (forward primer, FSeq2, sequence: TCGATGAAGAACGCAGCG; reverse primer, RSeq (Heisel et al., 2015)) and KAPA HiFi Hotstart plus dNTPs (Roche) for amplification. For PCR, 35 cycles were used for ITS2 amplicon generation from all sample types and for 16S amplicon generation from breastmilk; 25 cycles of PCR were used for 16S amplicon generation from infant feces. Breastmilk samples produced an average of 29,762 bacterial sequences/sample and 7,162 fungal sequences/sample; infant feces produced an average of 34,286 bacterial sequences/sample and 6,191 fungal sequences/sample. Amplicons were sequenced on an Illumina MiSeq system (Illumina, San Diego, CA) using V2 2×250 bp paired end chemistry. Bacterial and fungal amplicons were each sequenced on a single sequencing run. Sequences were deposited onto Minnesota Supercomputing Institute (University of Minnesota, Minneapolis, MN) servers for storage and analysis. All sequencing runs included negative control lanes and verified a lack of extraneous bacterial and fungal DNA contamination. In addition, for fungal sequencing, a synthetic “mock” fungal community (Palmer et al., 2018) was included for sequencing and verified that there was no “spill-over” of fungal DNA into adjacent sequencing lanes.

### Sequencing quality control and sequence alignment

Raw sequences were processed with the Shi7 (Al-Ghalith et al., 2018) version 0.9.9 learning program, which determined optimal parameters for processing. Sequences were then processed through Shi7, where they were trimmed, filtered by quality scores, and stitched per the optimized conditions determined by the learning program. Amplicon sequences from all samples were multiplexed into a single fasta file for downstream processing. Raw sequences are available in BioProject PRJNA880162 at the National Center for Biotechnology Information.

Quality controlled amplicon sequences were aligned to reference databases using BURST (Vangay et al., 2019; Al-Ghalith and Knights, 2020) version 0.99.7 f. To align bacterial sequences, a BURST reference database was generated from the 16S RefSeq collection compiled by the National Center for Biotechnology Information (NCBI) and accessed on July 4, 2017. Similarly, to align fungal sequences, a reference database was generated from the ITS RefSeq collection compiled by the NCBI and accessed on August 8, 2018. For all types of sequence alignment, BURST was used with a 95% identity cutoff flag and with the forward/reverse complement flag activated. The resulting.b6 files were converted to reference and taxonomy tables using embalmulate with “GGtrim” activated. Of note, sequencing reads of two fungal species, *Paecilomyces dactylethromorphus* and *Fusarium equiseti*, were identified in almost all samples, including multiple negative control samples. Because sequence data was generated from amplicons generated by PCR, it is not possible to determine exactly how much of either of these fungal taxa is a contaminant versus actually present in samples, thus, their data was not deleted from the sequencing files. As such, statistically significant results concerning either of these two taxa should be interpreted with caution. No other microbial sequences were identified in negative control samples.

### Statistical analyses and data visualization

Taxonomy and reference tables were imported into RStudio (2016) for analysis. The vegan, ape, nlme, ggplot2, ggbeeswarm, tidyverse, textshape, reshape2, randomcoloR, limma, ggsignif, Rtsne, igraph, gtools, and BiodiversityR libraries were used for data analysis, cohort comparisons, and figure production.

Statistical comparisons of clinical and demographic variables among cohort groups (as described in “Results”) were performed using analysis of variance (ANOVA), linear regression models, chi-squared analyses, and *t*-tests, as appropriate. The most abundant taxa were determined by ranking of the relative abundance percentages (defined as the sums of relative abundances of individual taxa across all samples), while the most prevalent taxa were determined by summing and then ranking the number of samples containing each taxon. Taxonomic counts from each sample were normalized using a centered log ratio (CLR) transformation. False discovery rate (FDR) correction using the Benjamini-Hochberg procedure was applied when multiple hypothesis testing was used with a threshold of FDR-corrected *value of p* (q) < 0.25 considered significant. For comparison of microbial alpha diversity measures, linear mixed-effects models included potentially relevant covariables (listed in Supplementary Tables S1, S3), as indicated in the “Results”. Statistical models also included a random effect for subject number as appropriate to account for repeated sampling (at 1 and 6 months) from the majority of infants. Relative taxa abundance comparisons were performed using Wilcoxon rank sum tests. Due to the multiple comparisons being performed, FDR-corrected *p* values (q values) were calculated. Beta diversity distances between samples were calculated using Euclidean dissimilarity indices on the CLR transformed data (Aitchison’s distance). Principal coordinates analysis (PCoA) and permutational analysis of variance (PERMANOVA, including covariables as listed in Supplementary Tables S1, S3) of beta diversity distances were used to visualize and calculate differences between cohorts with respect to microbial community structures. Procrustes analysis (vegan version 2.5.6, R version 3.4.3; Gower, 1975) was used to determine spatial similarity between coordinates in multidimensional space. Monte Carlo *value of p*s for Procrustes analyses are reported based on 1,000 permutations. Co-abundance correlations and interkingdom networks were constructed using bacterial and fungal sequences obtained after sequencing quality control procedures. In addition, samples were only included in the network analyses if subjects had paired bacterial and fungal sequence data for each specific sample type and time. Microbial (bacterial and fungal) taxa that were present in at least 30% of samples were included for each analysis. Spearman’s correlations were used to determine relationships between the (relative) abundances of bacterial and fungal taxa. For visualization purposes, correlations with an uncorrected *value of p* of less than 0.05 were used. Networks were visualized and neighborhood sizes were calculated using the igraph package, version 1.2.4 in R. Briefly, significant correlations were converted into an igraph object, and the Fruchterman-Reingold algorithm (Fruchterman and Reingold, 1991) was used to generate the network layout. Network plots were generated with ggplot2 version 3.3.5 and add2ggplot version 0.3.0; green coloring indicates a positive correlation while blue coloring indicates a negative correlation. Relative connectedness of each network was determined by dividing the total number of significant edges (Spearman’s correlation *p* < 0.05) by the total number of nodes, so higher values indicate greater connectedness.

## Results

### Study cohort consists of healthy mothers and infants involved in exclusive breastfeeding

As per the inclusion criteria, participants were healthy (i.e., no extended hospital stay beyond the birth hospitalization, or new diagnosis of major illness or disease during the first 6 months post-partum for either mother or infant), and all were involved in exclusive breastfeeding. In general, demographic and clinical factors did not differ among the three sample types (breastmilk, 1-month feces, 6-month feces; ANOVA, Supplementary Tables S1, S2, S4, S5), except for antibiotic exposure variables (Supplementary Table S4, see analysis below), indicating that differences in study population among sample types is not significantly changing the study cohort with respect to associated clinical features. Maternal pre-pregnancy BMI ranged between 18 and 40 (mean ~ 26.6). Numbers of male and female infants in the cohort were similar, most were delivered vaginally (~85%), and infant gestational age at birth was between 37.0–42.1 weeks (mean ~ 39.8). A majority of women reported this to be their first or second pregnancy (83%). Most subjects reported as White (~80% of women and infants), with the next most frequently reported category being Black or African American (~7% of women and infants). Only 1% of women and 3% of their infants self-identified as Hispanic. Exposure to prenatal antibacterial antibiotics occurred for ~22% of dyads. A substantial number of women also received perinatal and postnatal antibacterial antibiotics, potentially affecting 15–30% of samples, depending on the timing of antibiotic exposure and sample type. Two percent (2%) of infants were directly administered antibacterial antibiotics during the first month of life, and ~ 13% of infants between 1 and 6 months of age. Approximately 5% of women reported exposure to prenatal antifungal agents (topical, single treatment courses). Postnatal antifungal exposure was documented for approximately 10% of infants. Information regarding postnatal maternal antifungal exposure was not available for this cohort.

### Microbial communities in breastmilk are distinct from those of infant feces but share compositional features

**Figure 1:**
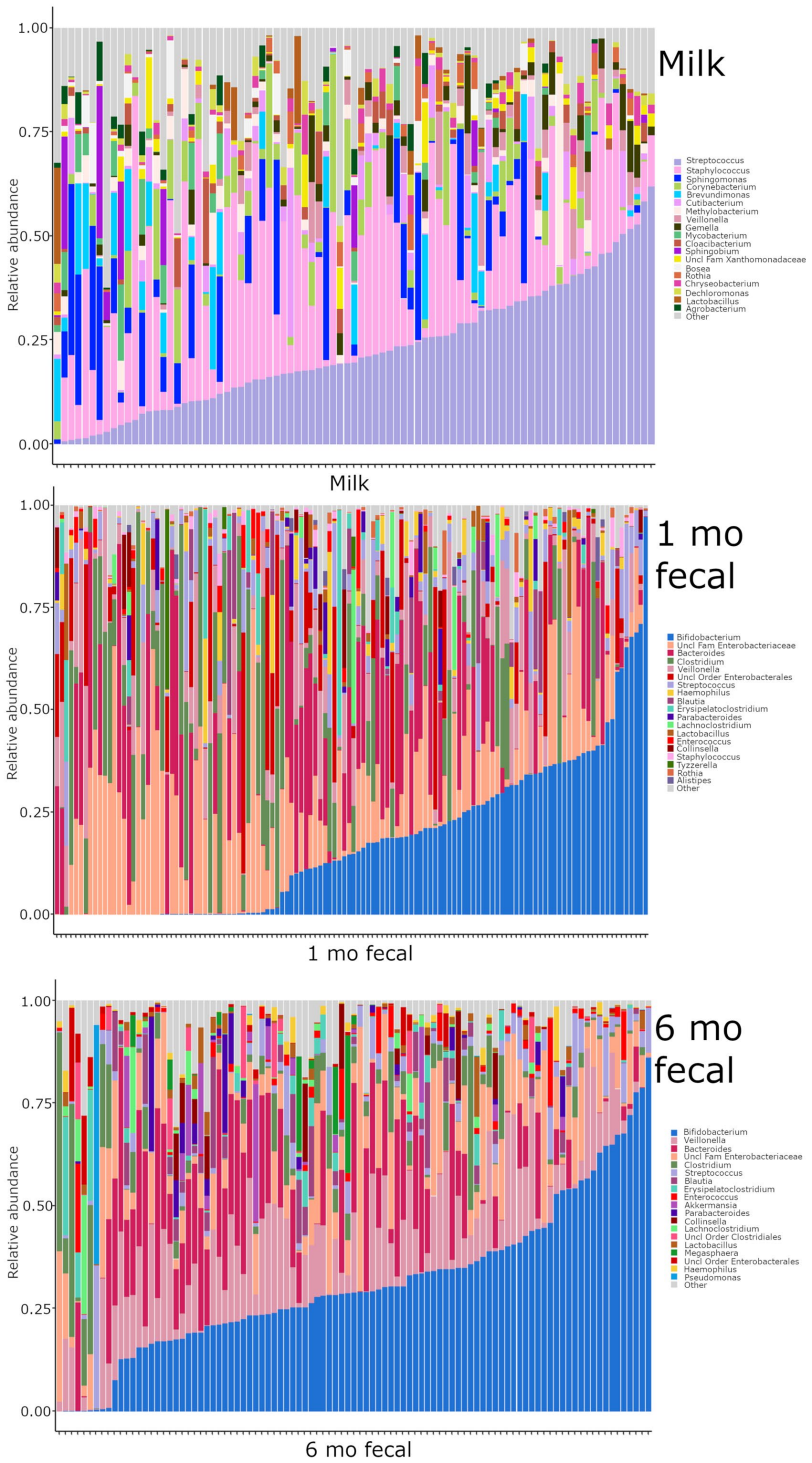
Relative abundances of bacterial taxa within each sample type. Each bar represents an individual sample, and samples are ordered by subject. “Other” represents taxa whose relative abundance is minimal as compared to the taxa included on the plot. Samples: *n* = 85 for breastmilk, 124 for 1-month feces, and 97 for 6-month feces.

**Figure 2:**
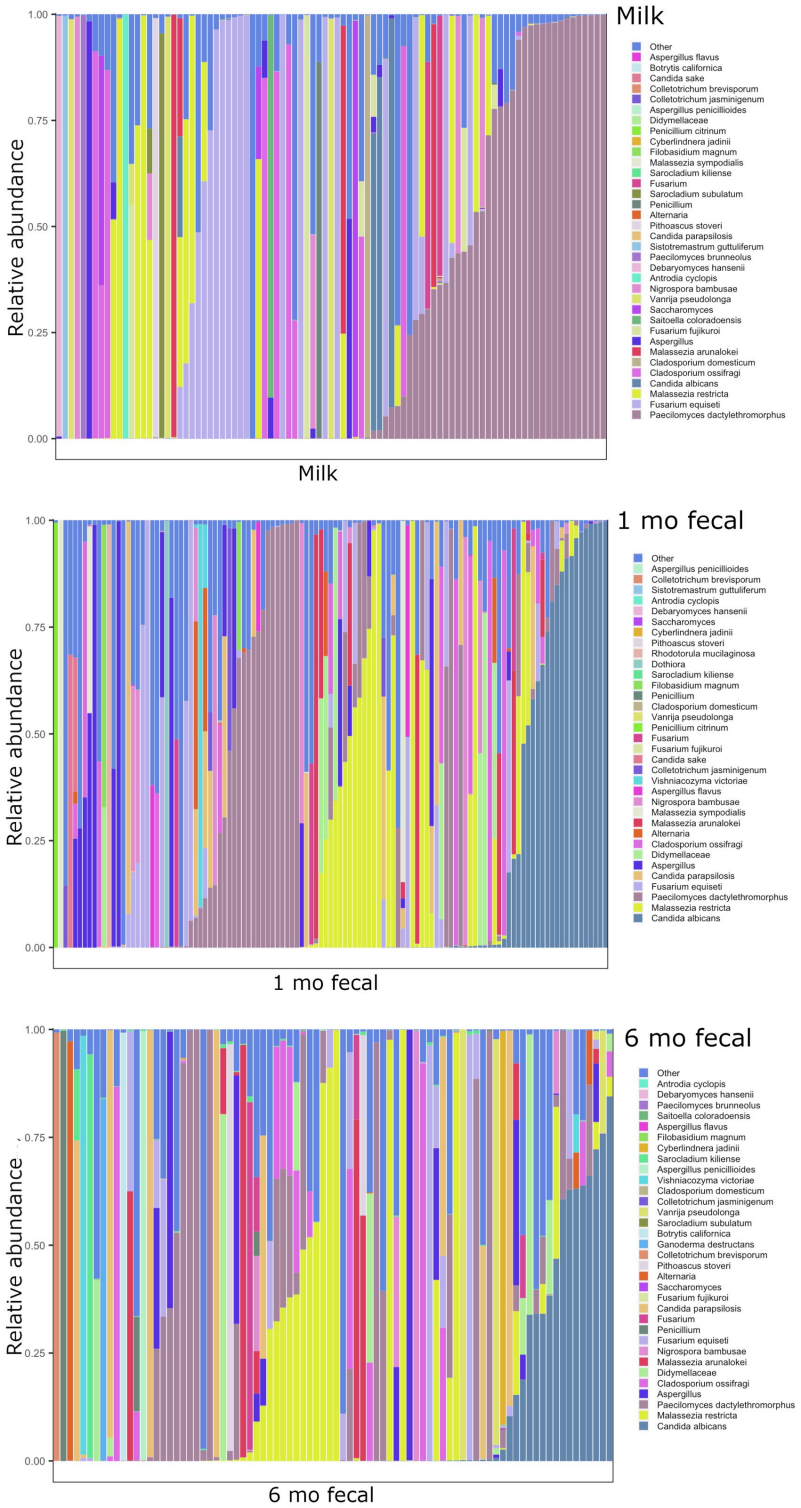
Relative abundances of fungal taxa within each sample type. Each bar represents an individual sample, and samples are ordered by subject. “Other” represents taxa whose relative abundance is minimal as compared to the taxa included on the plot. Samples: *n* = 91 for breastmilk, 115 for 1-month feces, and 84 for 6-month feces.

**Figure 3:**
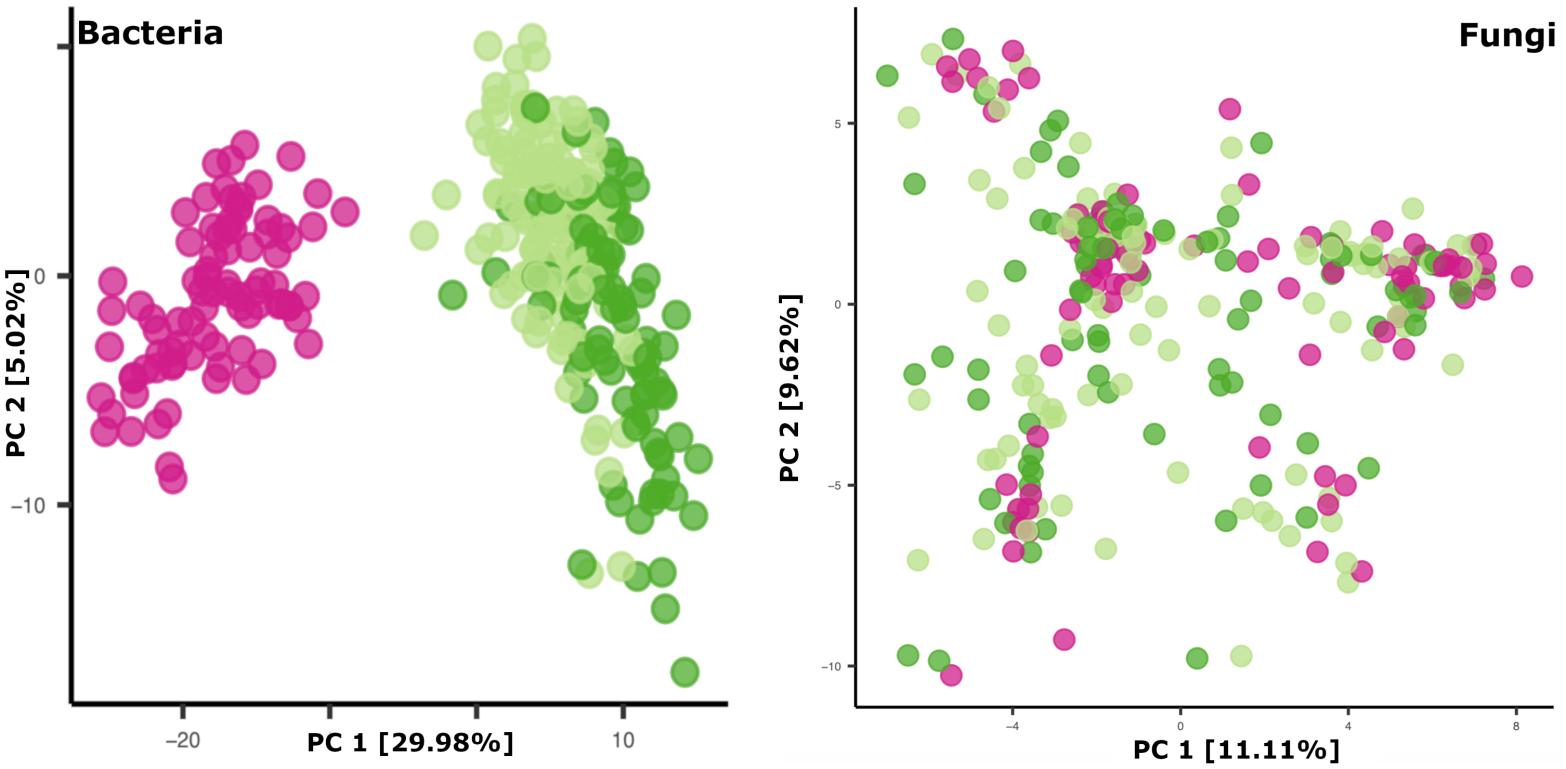
Principal coordinates plots of microbial beta diversity in breastmilk (pink dots), 1-month feces (light green dots), and 6-month feces (dark green dots). Bacterial community data points (left panel): *n* = 85 for breastmilk, 124 for 1-month feces, and 97 for 6-month feces. Fungal community data points (right panel): *n* = 91 for breastmilk, 115 for 1-month feces, and 84 for 6-month feces.

Breastmilk and infant feces had distinct bacterial (Figure 1), as well as fungal (Figure 2), community structures (PERMANOVA, *p* ≤ 0.002 for both bacteria and fungi, Figure 3). In addition, bacterial, but not fungal, fecal communities differed with respect to infant age (PERMANOVA, *p* = 0.001, Figure 3). The two most abundant and prevalent bacterial genera observed in breastmilk were *Streptococcus* and *Staphylococcus*, with both genera being found in all samples (Figure 1; Supplementary Table S7). *Bifidobacterium* was the most abundant and prevalent genus observed in both 1- and 6-month infant feces and *Streptococcus* was also highly abundant and prevalent (Figure 1; Supplementary Table S7). *Bifidobacterium* was also observed in breastmilk, although was not in the most abundant or prevalent bacterial taxa groups (mean abundance of 208 reads/sample, found in 31 of 85 milk samples). The most abundant and prevalent fungal species observed in breastmilk were *Paecilomyces dactylethromorphus, Fusarium equiseti*, *Malassezia restricta*, and *Candida albicans* (Figure 2; Supplementary Table S8). Like milk, the most abundant and prevalent fungal species in infant feces included *P. dactylethromorphus*, *M. restricta*, and *C. albicans*, at both 1 and 6 months of age (Supplementary Table S8). In addition, *Candida parapsilosis* was also highly abundant and prevalent in infant feces at both 1 and 6 months of age.

To further explore if related mothers and infants share microbial community features, the average beta diversity distances of microbial communities from related versus unrelated mother-infant pairs were compared. Overall, the distance for each related dyad (n = 70) was compared to ~70 unrelated mother-infant distances in a randomly generated permutation format. For 1-month feces, infant microbiomes and mycobiomes were not more similar to those of their own mother’s breastmilk as compared to those of unrelated mothers’ breastmilk (Wilcoxon rank sum tests of beta-diversity distances, *p* > 0.05).

### Clinical and socio-demographic associations with microbial community features in exclusively breastfeeding dyads

Previous studies have reported maternal and infant microbiome differences associated with maternal BMI, maternal and infant antibiotic exposure, and birth mode. We explored associations of these factors with microbiome and mycobiome features in dyads participating in exclusive breastfeeding. In this study, we did not observe microbiome or mycobiome variations in association with maternal pre-pregnancy BMI (data not shown) but did detect differences associated with antibiotic exposure and birth mode.

**Figure 4:**
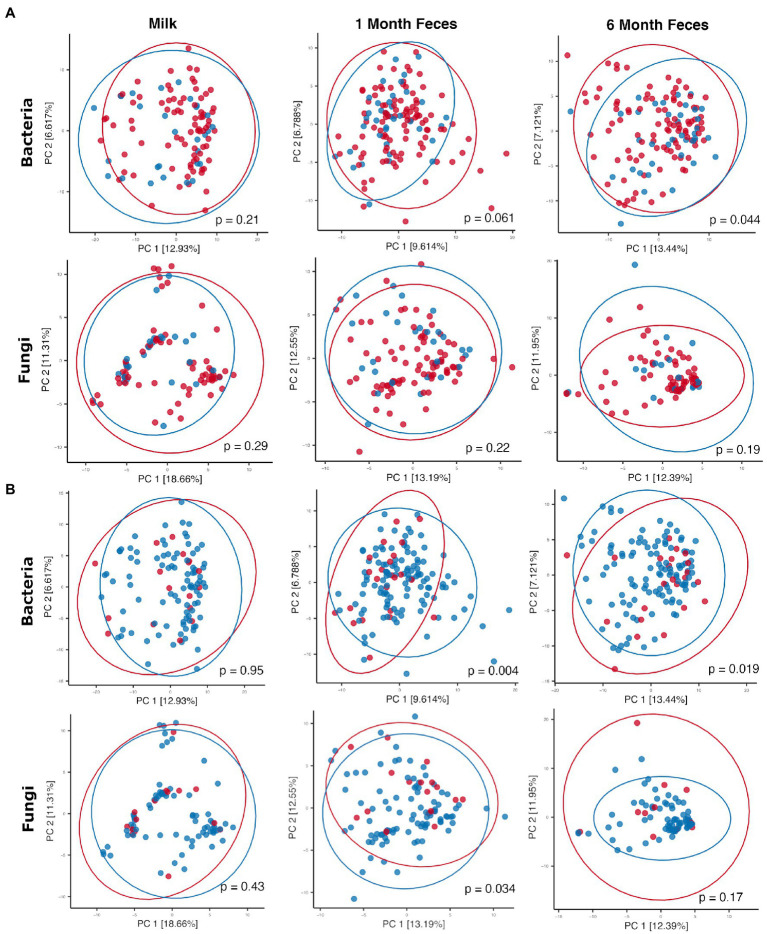
Principal coordinates plots comparing microbial communities by **(A)** perinatal antibiotic exposure (red dots = no exposure, blue dots = positive exposure) and **(B)** birth mode (red dots = Cesarean section, blue dots = vaginal birth). Ellipses indicate normal probability contours. For **A**, bacterial community data points (top row): *n* = 84 for breastmilk, *n* = 122 for 1-month feces, and *n* = 95 for 6-month feces; fungal community data points (bottom row): *n* = 90 for breastmilk, *n* = 112 for 1-month feces, and *n* = 83 for 6-month feces. For **B**, bacterial community data points (top row): *n* = 84 for breastmilk, *n* = 122 for 1-month feces, and *n* = 95 for 6-month feces; fungal community data points (bottom row): *n* = 90 for breastmilk, *n* = 112 for 1-month feces, and *n* = 83 for 6-month feces.

Antibiotics, by definition, have the potential to substantially modulate human microbial communities. As such, we included antibiotic exposure variables (as listed in Supplementary Table S3) in statistical models by sample type to understand if and how they were associated with microbial community variation in this cohort (results summary in Supplementary Table S9). Prenatal antimicrobial (antibacterial and antifungal) antibiotics had no associations with microbiome or mycobiome feature (alpha and beta diversity) variation, in either breastmilk or infant feces (both time points). In contrast, perinatal as well as postnatal maternal and infant antibacterial exposures were associated with microbiome variation as follows. Perinatal antibacterial exposure was associated with bacterial beta diversity differences in 6-month feces (Figure 4A) using statistical models that adjusted for the other antibiotic exposure variables listed in Supplementary Table S3. Microbiome differences were also observed in association with perinatal antibiotics in 1-month feces but did not meet statistical significance (PERMANOVA, *p* = 0.061) (Figure 4A). No association between perinatal antibiotics and breastmilk microbiome variation was observed. Postnatal maternal antibacterial exposure was associated with (higher) bacterial alpha diversity differences in one-month infant feces (mixed effect linear model, *p* = 0.04), whereas postnatal infant antibacterial exposure was associated with bacterial beta diversity differences in breastmilk (PERMANOVA, *p* = 0.02). Mycobiome variation was observed only for infant postnatal antibacterial exposure in 6-month-olds where exposure to antibiotics postnatally was associated with higher fecal fungal alpha diversity as compared to infants not exposed (mixed effect linear model, *p* = 0.04). Exposure to antifungal agents (only assessed prenatally) was not associated with significant bacterial or fungal community feature variation in this cohort.

Birth mode groups did not differ with respect to the majority of clinical covariables, except for maternal age (breastmilk samples only, younger for vaginal delivery) and, as noted above, perinatal antibacterial exposure (for all sample types, less exposure to antibiotics with vaginal delivery) (Supplementary Table S5). Statistical models comparing microbiome features by birth mode were adjusted for the clinical variables listed in Supplementary Table S5; these did not contribute to microbiome differences reported below, including perinatal antibiotic exposure and maternal age (*p* > 0.05 for all features in all models). For breastmilk, birth mode was not associated with variation in microbiome or mycobiome features (alpha [linear mixed effect models, Wilcoxon ranked sum tests] and beta [PERMANOVA] diversity, Supplementary Table S9; Figure 4B). For infant feces, bacterial alpha diversity did not differ by birth mode at 1 month of age but was higher in vaginally delivered infants at 6 months of age (Wilcoxon rank sum test, Shannon *p* = 0.006 [95% confidence interval = −0.57 to −0.09] and Simpson *p* = 0.006 [95% confidence interval = −0.15 to −0.02]). In addition, fecal bacterial taxonomic composition differed by birth mode, both at 1 month and at 6 months of age (PERMANOVA, *p* < 0.05, Figure 4B). Overall, the relative abundances of individual bacterial taxa in fecal microbiomes did not significantly differ by birth mode. With respect to fungi, fecal alpha diversity did not differ by birth mode at either 1 or 6 months of age. Mycobiome taxonomic composition differed by birth mode at 1 month but not at 6 months of age (PERMANOVA, Figure 4B). Of note, for 1-month feces, the relative abundance of the skin-associated fungus *M. restricta* was significantly higher for infants born by Cesarean section as compared to those born vaginally (Wilcoxon rank sum test, q = 0.14). This association was not observed in 6-month feces. Differential abundances by birth mode were not observed for any other fungal taxa in feces at either time point.

Self-identified race has been associated with gut microbiome variation in adults (Brooks et al., 2018) and infants (Pannaraj et al., 2017). In the current cohort, infant race was associated with microbiome variation in 1-month (beta diversity PERMANOVA, *p* = 0.01), but not 6-month feces. For breastmilk, maternal race was not associated with microbiome variation, although bacterial counts differed between groups (Welch’s *t*-test, *p* = 0.03, CI [−1.76, −0.09]. Mycobiome feature differences were not observed between the two race groups for any sample type. Race groups (both maternal and infant) differed with respect to infant gestational age at birth, but no other clinical variables listed in Supplementary Tables S1 and S3 (Chi-squared tests, *p* > 0.05 for all). We also compared race groups with respect to demographic and behavioral factors including the HEI 2015, a measure of adherence to a healthy diet, maternal education, and family income. Maternal education and household income were both higher for Group 2 (White) as compared to Group 1 (all other self-identified races) (Chi-squared tests, *p* < 0.05 for both factors and when testing either maternal or infant race) and HEI did not differ between the groups (*p* > 0.05). However, although they differed in level or frequency by race, infant gestational age, maternal education, and family income were not associated with microbiome variation in any sample type.

### Interkingdom relationships in breastmilk and infant feces

**Figure 5:**
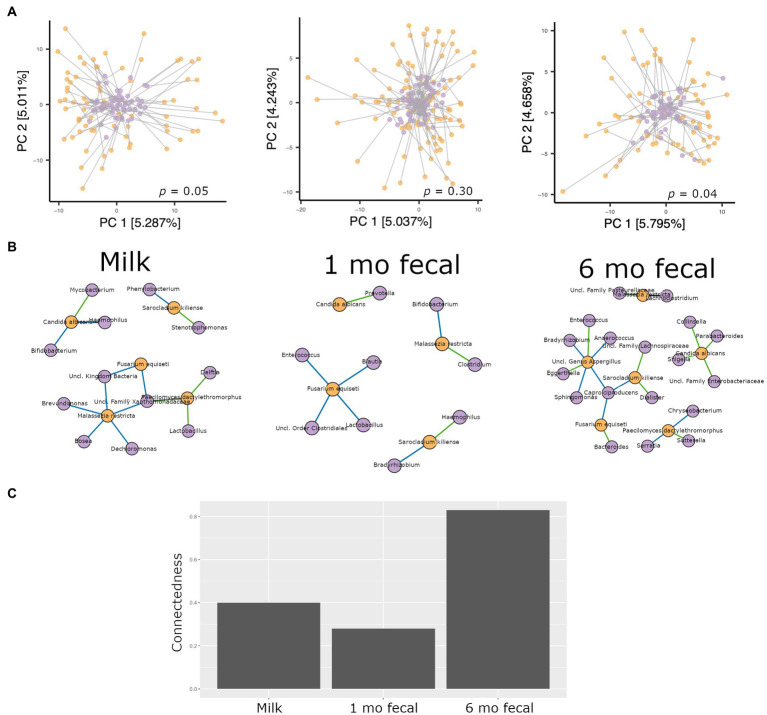
Analyses of interkingdom relationships in breastmilk (left panels), 1- and 6-month feces (middle and right panels, respectively). **(A)** Procrustes analysis comparing the spatial fit of unweighted Unifrac principal coordinate matrices of bacterial communities (purple spheres) and Bray-Curtis principal coordinate matrices of fungal communities (orange spheres) for each sample type. Breastmilk *n* = 75, 1-month feces *n* = 100, 6-month feces *n* = 69. **(B)** Interkingdom network maps of interactions between bacterial (purple spheres) and fungal (orange spheres) taxa by sample type (Breastmilk *n* = 75, 1-month feces *n* = 100, 6-month feces *n* = 69. Blue line, negative correlation; green line, positive correlation. **(C)** Interkingdom connectedness values (see Methods) for each sample type.

Procrustes analysis was used to evaluate relationships between bacterial and fungal communities in breastmilk as well as in infant feces. In feces from 1-month-old infants, microbiomes and mycobiomes lacked congruency, that is, their phylogenetic structures were not correlated. However, congruence of bacterial and fungal communities was observed for breastmilk as well as 6-month feces (Figure 5A), suggesting mature communities in breastmilk and co-maturation of the two kingdoms over time in the infant gut.

Network analysis to identify interkingdom co-abundance relationships (of bacterial and fungal taxa present in more than 30% of the samples) was also performed for microbes in breastmilk and infant feces. For network comparisons, edges with uncorrected significant correlations (Spearman’s correlation, *p <* 0.05) were considered. Multiple co-abundance correlations were identified in breastmilk, as well as in infant feces at both time points (Figure 5B). No specific bacterial-fungal taxa correlations were shared among sample types. Of note, the probiotic-associated bacteria, *Bifidobacterium*, was negatively correlated with *C. albicans* in breastmilk and with *Malassezia restricta* in 1-month infant feces. To investigate how connected bacterial and fungal communities were to each other within the networks, interkingdom connectedness values were calculated for each sample type. Although there were no statistically significant differences in interkingdom network connectedness among breastmilk, 1-month and 6-month feces (pairwise *t*-tests, *p* > 0.05), the relative connectedness of the 6-month fecal network was generally higher than that at 1 month (0.83 significant interactions/node vs. 0.28 significant interactions/node, Figure 5C) suggesting that a richer and more complex interkingdom network develops in the infant gut over time.

**Figure 6:**
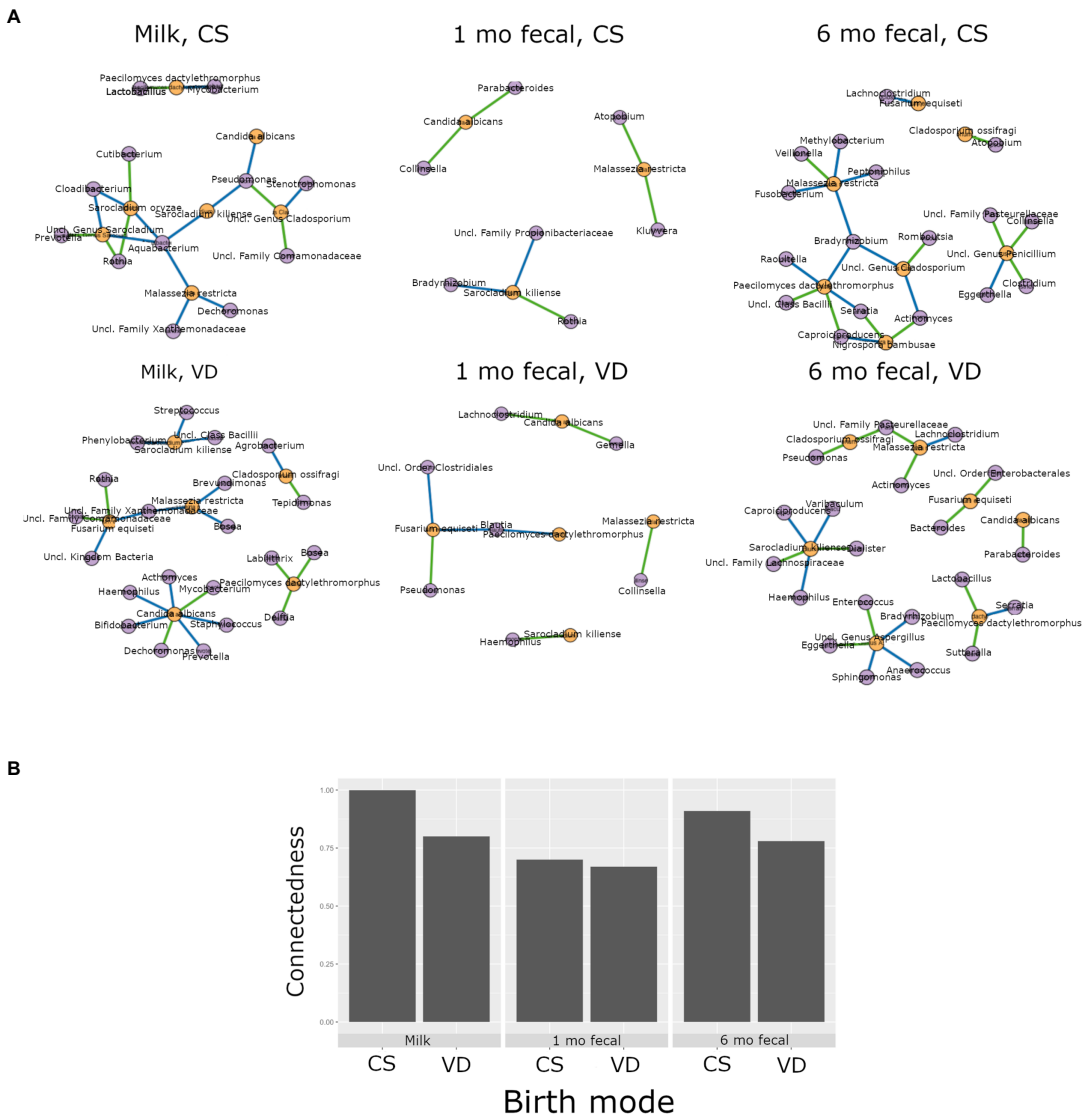
Interkingdom relationship comparisons by birth mode. Panels in **A** are interkingdom network maps of significant interactions between bacterial (purple) and fungal (orange) taxa and are organized by sample type. Blue line, negative correlation; green line, positive correlation. Plot in **B** is of interkingdom connectedness values (see Methods) by sample type. Sample n’s included in network analyses for Cesarean section delivery (CS, top row): breastmilk 12, 1-month feces 15, 6-month feces, 9 and for Vaginal delivery (VD, bottom row): breastmilk 62, 1-month feces 83, 6-month feces 59.

**Figure 7:**
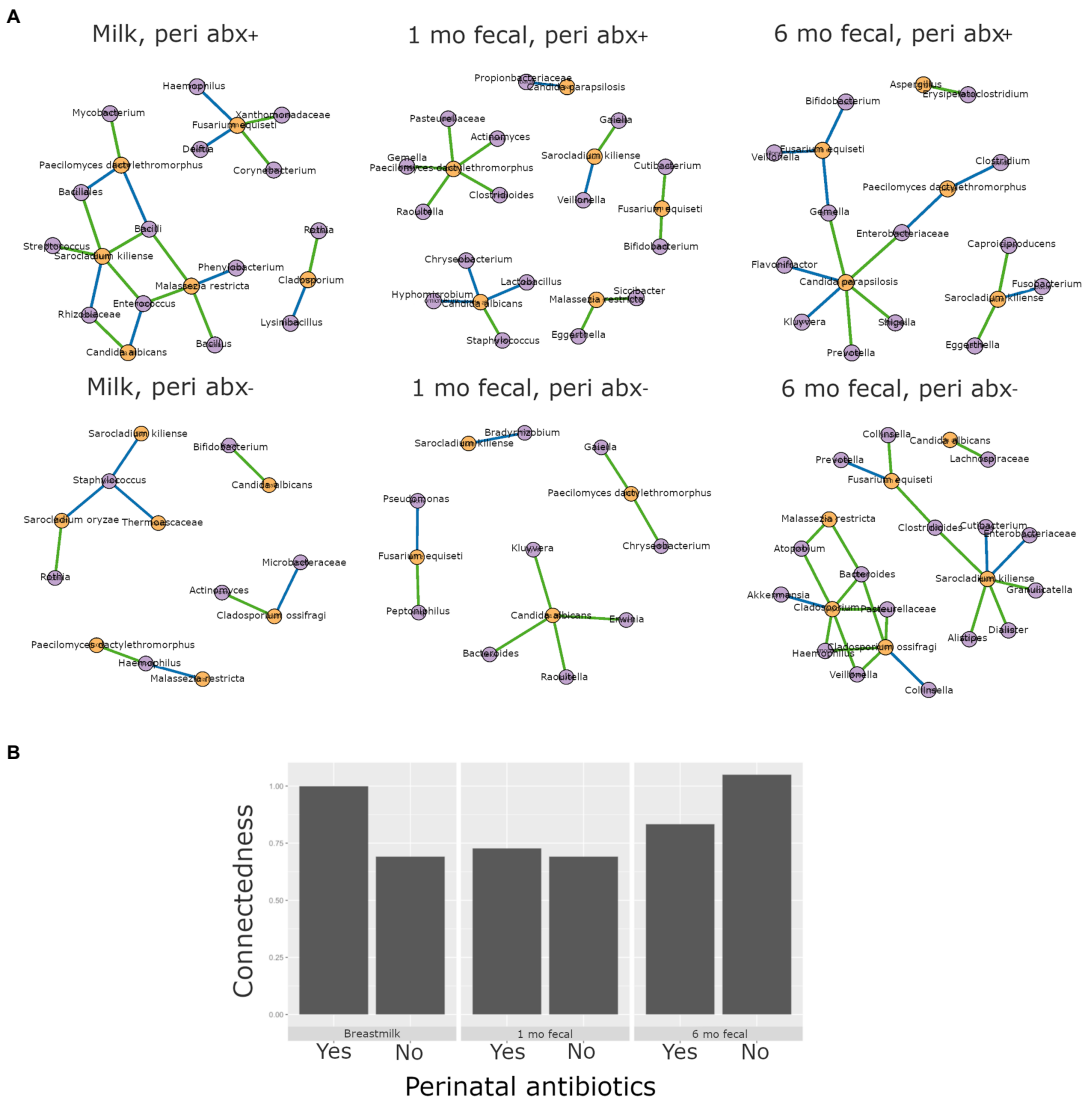
Interkingdom relationship comparisons by perinatal antibiotic exposure. Panels in **A** are interkingdom network maps of significant interactions between bacterial (purple) and fungal (orange) taxa and are organized by sample type. Blue line, negative correlation; green line, positive correlation. Plot in **B** is of interkingdom connectedness values (see Methods) by sample type. Sample n’s included in network analyses for Perinatal antibiotic-exposed (peri abx +, top row): breastmilk 19, 1-month feces 26, 6-month feces 18 and for Perinatal antibiotics unexposed (peri abx -, bottom row): breastmilk 55, 1-month feces 72, 6-month feces 62.

**Figure 8:**
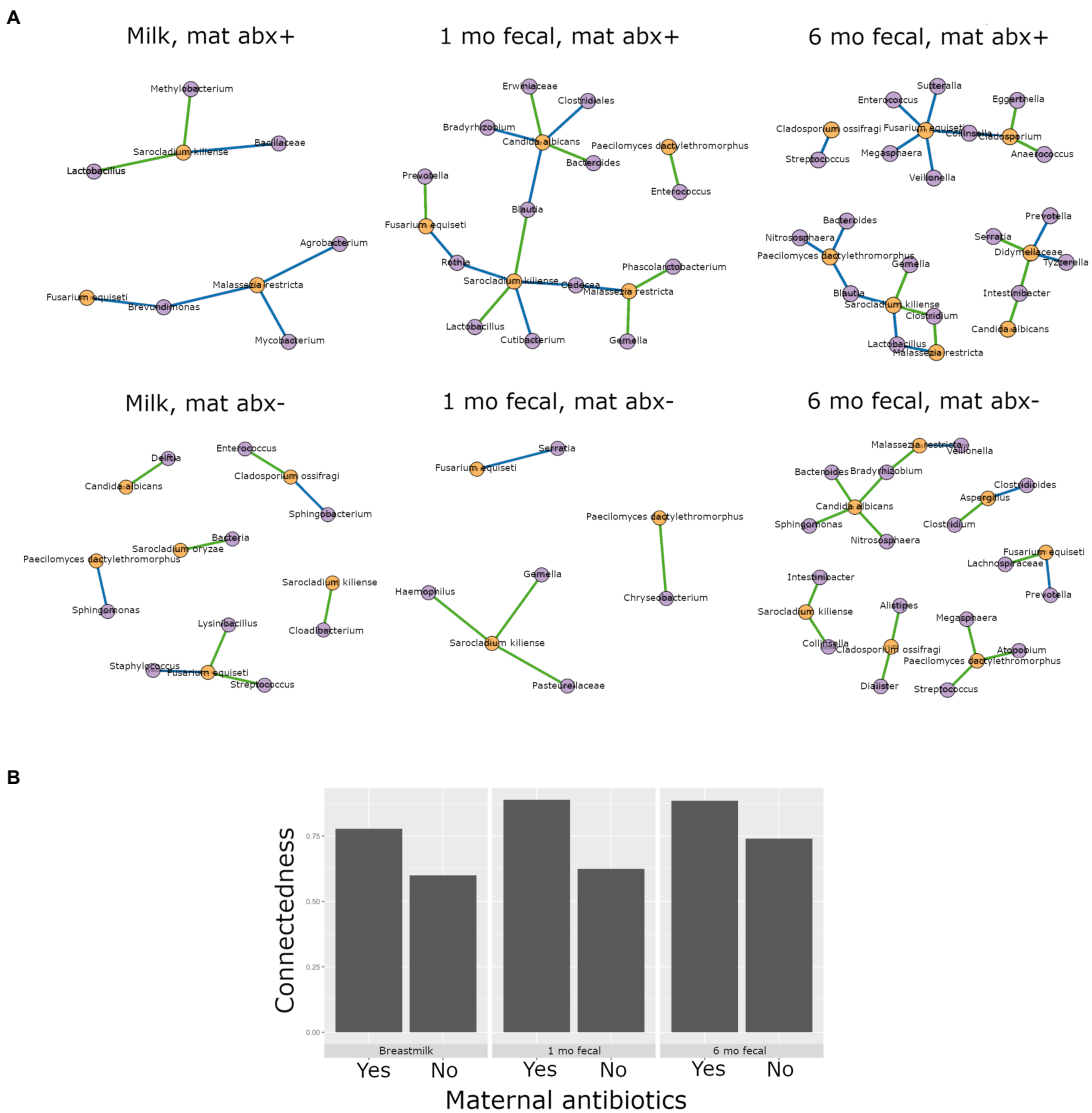
Interkingdom relationship comparisons by maternal postpartum antibiotic exposure. Panels in **A** are interkingdom network maps of significant interactions between bacterial (purple) and fungal (orange) taxa and are organized by sample type. Blue line, negative correlation; green line, positive correlation. Plot in **B** is of interkingdom connectedness values (see Methods) by sample type. Sample n’s included in network analyses for Maternal postpartum antibiotic exposed (mat abx +, top row): breastmilk 15, 1-month feces 24, 6-month feces 13 and for Maternal postpartum antibiotic unexposed (mat abx -, bottom row): breastmilk 60, 1-month feces 76, 6-month feces 69.

Higher interkingdom network connectedness was also observed when samples were analyzed by birth mode and antibiotic exposure (summary of all values in Supplementary Table S10). Breastmilk and infant feces (1- and 6-months) all tended toward higher connectedness for dyads delivering by Cesarean section as compared to those delivering vaginally (Figure 6B). In general, higher bacterial-fungal connectedness was also observed to be associated with perinatal antibiotics for breastmilk and 1-month infant feces (Figure 7B), as well as with maternal postnatal antibiotics for breastmilk, 1-month, and 6-month infant feces (Figure 8B). The trend towards higher interkingdom connectedness with increasing age in infant feces continued to be observed in birth mode and antibiotic exposure sub-cohorts, except for those exposed to maternal postpartum antibiotics. With respect to specific bacteria-fungus co-abundance relationships, very few interactions were shared among sample types for either birth mode or antibiotic exposure subgroups. Of note, a negative correlation was again observed for *Bifidobacterium* and *C. albicans* in breastmilk when interkingdom networks were compared by birth mode, with this relationship being found only in the milk of dyads who delivered vaginally (Figure 6A). In contrast, *Bifidobacterium* and *C. albicans* were positively correlated in breastmilk of mothers, but only among those not exposed to perinatal antibiotics (Figure 6A).

## Discussion

This study is one of the few to our knowledge that describes interkingdom bacterial-fungal relationships during early-life development of human microbial communities. We identified bacterial-fungal correlations in breastmilk and in 1- and 6-month infant feces. Interkingdom analyses revealed that maturation of bacterial-fungal networks appears to develop over time, with richer, more complex networks of higher congruency observed in older infants, consistent with a prior study reporting a positive correlation between interkingdom network complexity and gestational age in the premature infant gut (Willis et al., 2019). In subgroup analyses, Cesarean section delivery and antibacterial antibiotic exposure (perinatal and maternal post-partum) all tended to be associated with higher interkingdom connectivity in all sample types. The exception to this was for perinatal antibiotics for 6-month feces. We propose that the contribution of perinatal antibiotics to infant gut microbiome structure may be negligible by 6 months of age. Overall, the finding that both Cesarean section birth and antibacterial antibiotics were associated with higher interkingdom connectivity indicates altered development of bacterial-fungal relationships in these clinical scenarios. In addition, to these interkingdom results, we observed that antibacterial antibiotic exposure was associated with changes to fungal community structures. Six-month-old infants exposed to postnatal infant antibiotics had higher fungal diversity than unexposed infants. This trans-kingdom effect, indicating that modulation of bacterial communities is associated with fungal community variation, is consistent with a prior study (Ventin-Holmberg et al., 2022) that reported higher fungal alpha diversity in the feces of infants exposed to postnatal antibacterial antibiotics. The converse trans-kingdom relationship (antifungal association with bacterial microbiome variation) has been reported in mice (Heng et al., 2021) but was not observed in the current study or in any other study, to our knowledge, in humans.

Our analyses of both bacteria and fungi, and their interactions, in mothers and their infants begin to fill a knowledge gap with respect to early life human microbiome development. Altered gut bacterial-fungal interkingdom relationships have been identified in adults with mental health disorders (Jiang et al., 2020), *Clostridium difficile* infection (Stewart et al., 2019), and preceding the diagnosis of invasive candidiasis in patients who received allogeneic hematopoietic stem cell transplants (Zhai et al., 2020). Similar investigations for associations in the infant gut have not yet been described, but emerging research in animals reveals a role for early life interkingdom interactions in long-term health outcomes. For example, Boutin and colleagues found that fungi interact with bacteria in the neonatal murine gut (*via* bacterially derived short chain fatty acids) to influence the severity of allergic airway disease later in life (Boutin et al., 2021a).

This study focused on the analysis of microbiomes and mycobiomes of mother-infant dyads who were involved in exclusive breastfeeding. This aspect of the cohort design allowed us to identify connections between infant gut microbial communities to those from their sole source of enteral nutrition and a potential source of pre- and probiotics: their mothers’ breastmilk. Infant formula, as compared to breastmilk, is microbially deficient and its consumption is well known to be associated with altered infant gut bacterial microbiome composition (e.g., higher bacterial alpha diversity and lower relative abundance of Bifidobacterium) and functions that are important for immunity, carbohydrate metabolism, and antibiotic resistance (Di Guglielmo et al., 2022; Pärnänen et al., 2022). Thus, analysis of this exclusively breastfeeding cohort enabled the characterization of bacterial, fungal and interkingdom relationships in microbial communities of infants while minimizing dietary variation due to non-human milk (formula) feedings and other foods.

Breastmilk microbes are an important source of early life gut microbial communities (Pannaraj et al., 2017). In the current cohort, we found that bacterial and fungal community compositions of breastmilk were different from those of the infant gut. These results are consistent with the idea of niche-specific micro- and mycobiome development, which is supported by many human studies including those in infants (Dominguez-Bello et al., 2010; Pannaraj et al., 2017; Ward et al., 2018). Although the overall taxonomic compositions differed by sample type (breastmilk, 1-month fecal, 6-month fecal), common prevalent and abundant bacterial and fungal taxa (in particular, *Bifidobacterium*, *Streptococcus*, *Staphylococcus*, *Candida,* and *Malassezia* species) were observed in both breastmilk and infant feces. Bacterial and fungal communities of mothers and their infants were not more alike, however, as compared to unrelated mother-infant pairs in our cohort. With respect to bacteria, this result contrasts with previous reports (Pannaraj et al., 2017; Fehr et al., 2020) which showed that related dyad microbiomes (breastmilk and infant feces) were more like each other than those of unrelated mothers and infants. The lack of similarity between microbiomes of related dyads in our cohort may be due to the earlier time point of assessment (1 month of infant age) as compared to previous studies (majority when infant was over 3 months of age). It is possible that the synergy of milk and infant gut microbiomes becomes more established the longer the period of breastmilk exposure. Although this study was not designed to characterize longitudinal dynamics of milk and infant microbial communities, we did observe age-dependent differences for several bacterial as well as fungal microbiome features in the infant feces collected at 1 and 6 months, and their association with clinical and demographic characteristics. These age-dependent results align with previous studies (Schei et al., 2017; Stewart et al., 2018; Boutin et al., 2021b) reporting microbial community maturation during infancy and highlight the importance of including the age of the subject/sample as a covariable in the analysis of early life microbial communities.

Antibiotics are known to affect microbiome variation, depending on antibiotic timing, duration, and type. For pregnant women and infants, perinatal (at the time of birth) antibacterial exposure is extremely common [32% of women (Tita 2010)] and is associated with gut dysbiosis in infants during the first weeks of life (Wang et al., 2020). Postnatal antibacterial antibiotic exposure (including maternal antibiotic exposure *via* breastmilk) is also common in infants and children, with an estimated 10% of infants receiving antibiotics during the first week of life (Puopolo and Eichenwald, 2010; Escobar et al., 2014) and another 20–30% receiving prescriptions for antibiotics during outpatient primary care visits (Nyquist et al., 1998; Nash et al., 2002). These postnatal exposures have also been associated with infant gut dysbiosis, which usually recovers over a timespan of weeks to months (Jernberg et al., 2010; Fouhy et al., 2012; Dardas et al., 2014). In general, we observed relatively few associations between antibiotic exposure timing groups and microbiome feature variation in the current cohort. This result may be related to the lower numbers of infants exposed to postnatal antibiotics in our cohort as compared to previous studies or, alternatively, that exclusive breastmilk feedings are protective to infant gut dysbiosis as has been suggested by others (Azad et al., 2016). An exception to this was for perinatal antibiotic exposure (~25% of mother-infant dyads in our cohort), with a difference in beta diversity being observed in 6-month feces (*p* = 0.04), and a difference in 1-month feces, although this result did not meet statistical significance (*p* = 0.06). This finding adds to the growing number of reports of microbiome disruption in infants exposed to perinatal antibiotics, which has been associated with the development of antibiotic resistance, asthma and obesity (Aloisio et al., 2016; Nogacka et al., 2017; Neuman et al., 2018). For antifungal antibiotics, mycobiome feature variation was not observed for any sample type, but because the number of subjects exposed to antifungals in this cohort was small (<10%), our study may have been underpowered to detect differences. Although antifungal antibiotics have been associated with gut mycobiome variation and associated gut inflammation in animals (Wheeler et al., 2016; Heng et al., 2021), this has not yet been rigorously studied in humans.

Microbial transmission from mother to infant at the time of birth is an important mechanism involved in early gut microbiome establishment. Birth mode affects the composition of microbiomes transmitted to the infant gut, with some studies reporting differences (in bacterial communities) persisting up to 2 years of age (reviewed in (Wang et al., 2020)). We found, for the first time to our knowledge, that birth mode is associated with variation in infant gut mycobiomes at 1 month of age. Overall fungal community composition differed by birth mode and the relative abundance of the skin-associated fungus *M. restricta* was higher in the feces of infants delivered by Cesarean section. This observation is akin to that of our previous study (Ward et al., 2018) where fungi prevalent in the vagina (*C. albicans*) were found to be more abundant on the skin of infants delivered vaginally. Together, these results support the idea that birth mode/body site modulates early life mycobiomes. In our study cohort, Cesarean section birth was also associated with lower bacterial alpha diversity at 6 months of age as well as different overall bacterial community compositions (beta diversity) at both 1 and 6 months of age. Although reduced levels of Bacteroides have been observed in the feces of infants delivered by Cesarean section (Stewart et al., 2018; Shao et al., 2019b), we did not see this association in our cohort potentially due to differences in diet (other studies included dyads involved in non-exclusive breastmilk feedings), sampling times, cohort size, and/or DNA-related methodological variations as compared to those of the other studies. Of note, antibacterial antibiotics are almost universally administered to pregnant women at the time of Cesarean section to prevent surgical infections and, thus, could potentially contribute to microbial differences based on birth mode. Our results, obtained from multivariate linear modelling which included antibiotic exposure covariables, identified birth mode as an independent contributor to infant gut microbial variation. This is consistent with the findings reported using a mouse model which showed gut microbiome variation in pups delivered by Cesarean section and not exposed to antibiotics as compared to those delivering vaginally (Martinez et al., 2017).

This study had limitations. First, the taxonomic resolution obtained with16S amplicon sequences is lower than that obtained with ITS2 amplicon sequences. This results in bacterial taxonomic identification to no more specific than the genus level and fungal taxonomic identification to the species level, and thus, different taxonomic resolutions are being compared in interkingdom analyses. Second, we observed microbiome variation in association with race, which should be interpreted with caution. Race is a social, not biological or genetic, construct and as such, our study was limited in that we did not have extensive assessment of social factors at the individual and community levels that could help interpret these differences. Although we were able to consider maternal diet (HEI), maternal education and household income in the analyses, none of these were associated with microbiome variation, and we did not have additional information regarding markers of economic stability, neighborhood and physical environments, and community and social contextual variables that could contribute to microbiome variation. Studies with larger, more diverse subject cohorts with deeper data collection with respect to social, institutional, and systemic factors are needed to understand their contributions to microbiome variation in families.

## Summary

In summary, our study adds to a growing literature reporting interkingdom relationships in human microbial communities and underlines the importance of continued research to understand microbiome-mediated health effects beyond those of a single microbial kingdom. A more complete knowledge of human-associated microbiomes is particularly needed for the period of infancy, the important window of life when microbiomes co-develop with many aspects of human physiology to potentially influence health outcomes for the long term.

## Data availability statement

The datasets presented in this study can be found in online repositories at: https://www.ncbi.nlm.nih.gov/, PRJNA880162.

## Ethics statement

The studies involving human participants were reviewed and approved by Institutional review boards of the University of Oklahoma, University of Minnesota, and the HealthPartners Institute. Written informed consent to participate in this study was provided by the participants (pregnant women) for themselves and their infants.

## Author contributions

TH, AJ, KJ, DF, DK, ED, and CG contributed to the conception and design of the study. JH developed and maintained the database containing the clinical and demographic characteristics of the study cohort. TH, AJ, SG, AD, and ES analyzed microbial sequence data and performed statistical analyses for this work. TH, AJ, SG, AD, ES, DK, ED, and CG collaborated to interpret the data in this study. TH, AJ, SG, and CG wrote sections of the manuscript draft. JH, EN, SP, DF, and ED critically reviewed the manuscript and provided revisions for important intellectual content. All authors contributed to the article and approved the submitted version.

## Funding

The MILk Study which provided the cohort and milk samples for this study was supported by NIH/NICHD grant (R01HD080444) to ED and DF; microbiome and mycobiome analyses of cohort biospecimens was supported by a University of Minnesota Masonic Children’s Hospital Research Fund Award to CG, ED, and DK, a University of Minnesota Office of Academic and Clinical Affairs Faculty Research Development Grant to CG, ED, KJ, and DK and NIH grants (R21AI139730, R21HD099473) to CG and DK. EN was supported by NIH/NIDDK fellowship grant (T32DK083250) and K99/R00 K99HD108276.

## Conflict of interest

The authors declare that the research was conducted in the absence of any commercial or financial relationships that could be construed as a potential conflict of interest.

## Publisher’s note

All claims expressed in this article are solely those of the authors and do not necessarily represent those of their affiliated organizations, or those of the publisher, the editors and the reviewers. Any product that may be evaluated in this article, or claim that may be made by its manufacturer, is not guaranteed or endorsed by the publisher.

## Footnotes

^1^ ClinicalTrials.gov
