# Supplementary material for: Bacterial, fungal, and interkingdom microbiome features of exclusively breastfeeding dyads are associated with infant age, antibiotic exposure, and birth mode
Source: Front Microbiol. 2022 Nov 17;13:1050574. doi: 10.3389/fmicb.2022.1050574 (PMC9714262; doi:10.3389/fmicb.2022.1050574)
Supplement: Supplementary file 1 [file Data_Sheet_1.docx]

Supplemental Table 1. Clinical characteristics associated with samples analyzed from total cohort of mother-infant dyads.

|  | Breastmilk  (n = 85/91)^a^ | 1-month feces  (n = 124/115)^a^ | 6-month feces  (n = 97/84)^a^ | *p* values |
| --- | --- | --- | --- | --- |
| Birth mode,  % Cesarean | 16.7/16.7 | 14.8/17.0 | 13.7/14.4 | 0.85/ 0.87 |
| Pre-pregnancy BMI,  mean kg/m^2^±SD | 27.4±5.0/26.8±5.1 | 26.6±4.8/26.8±4.6 | 26.4±4.7/26.4±4.5 | 0.33/ 0.75 |
| Healthy Eating Index (HEI), mean±SD | 65.3±9.2/65.6±9.0 | 65.6±8.5/65.5±8.2 | 65.8±8.5/65.6±8.8 | 0.90/1.00 |
| Infant sex,  % female | 52.9/53.8 | 50.8/47.8 | 58.8/51.5 | 0.81/ 0.88 |
| Maternal age,  mean years±SD | 31.6±4.0/31.4±4.1 | 31.0±3.9/31.2±4.2 | 31.5±4.2/31.0±4.1 | 0.47/ 0.86 |
| Infant gestational age,  mean weeks±SD | 39.7±1.1/39.7±1.1 | 39.8±1.1/39.8±1.1 | 39.7±1.2/39.9±1.1 | 0.87/ 0.80 |
| Infant race,  % Group 1^b^ | 20.2/16.7 | 15.8/19.8 | 16.117.7 | 0.92/ 0.84 |
| Maternal race,  % Group 1^c^ | 19.0/16.7 | 13.9/15.9 | 14.7/13.3 | 0.59/ 0.80 |

^a^ number of samples included in analyses (indicated as bacterial sample n / fungal sample n) after quality control procedures after sequencing (see Methods). Due to differences in sequencing output for bacteria vs fungi, the number of samples for their analyses differed as did the Table data for each kingdom (bacterial-associated data/fungal-associated data).

^b^ The only race category not included in Group 1 is White. Group 1 infant race composition (subject n for bacterial/fungal analysis) by sample type: Breastmilk; American Indian/Alaska native 2/2, Asian 2/3, Black or African American 7/6, More than one race 1/1, Other 3/3. 1-month feces; American Indian/Alaska native 3/3, Asian 2/4, Black or African American 8/8, More than one race 3/3, Other 3/4. 6-month feces; American Indian/Alaska native 2/2, Asian 1/4, Black or African American 6/6, More than one race 3/3, Other 3/2.

^c^ Group 1 maternal race composition subject n for bacterial/fungal analysis) by sample type: Breastmilk; American Indian/Alaska native 2/2, Asian 2/3, Black or African American 7/6, More than one race 1/1, Other 4/3. 1-month feces; American Indian/Alaska native 3/3, Asian 3/3, Black or African American 7/7, More than one race 3/2, Other 1/3. 6-month feces; American Indian/Alaska native 2/2, Asian 1/3, Black or African American 5/6, More than one race 2/1, Other 4/1.

Supplemental Table 2. Clinical characteristics associated with subset of samples included in network analyses.

|  | Breastmilk (n = 75)^a^ | 1-month feces (n = 100)^a^ | 6-month feces (n = 69)^a^ | *p* value |
| --- | --- | --- | --- | --- |
| Birth mode, % Cesarean | 16.2 | 15.3 | 13.2 | 0.88 |
| Pre-pregnancy maternal BMI, mean kg/m^2^±SD | 26.3±4.9 | 26.8±4.7 | 26.4±4.6 | 0.72 |
| Infant sex, % female | 54.7 | 49.0 | 57.9 | 0.50 |
| Maternal age, mean years±SD | 31.5±4.0 | 31.1± 4.0 | 31.4±4.3 | 0.80 |
| Gestational age at birth, weeks±SD | 39.8±1.0 | 39.8± 1.1 | 39.8±1.2 | 0.97 |
| Infant race, % Group 1^b^ | 13.5 | 17.7 | 16.7 | 0.76 |
| Maternal race, % Group 1^c^ | 14.9 | 14.3 | 11.8 | 0.85 |

^a^ number of samples remaining after quality control procedures after sequencing and after including only samples with paired bacterial and fungal sequences (see Methods).

^b^ The only race category not included in Group 1 is White. Group 1 infant race composition for each sample type: Breastmilk; American Indian/Alaska native 1, Asian 3, Black or African American 5, More than one race 0, Other 1. 1-month feces; American Indian/Alaska native 3, Asian 2, Black or African American 7, More than one race 2, Other 3. 6-month feces; American Indian/Alaska native 2, Asian 1, Black or African American 4, More than one race 3, Other 1.

^c^ Group 1 maternal race composition for each sample type: Breastmilk; American Indian/Alaska native 1, Asian 3, Black or African American 5, More than one race 0, Other 2. 1-month feces; American Indian/Alaska native 3, Asian 2, Black or African American 6, More than one race 2, Other 1. 6-month feces; American Indian/Alaska native 2, Asian 0, Black or African American 4, More than one race, 1 Other 1.

Supplemental Table 3. Antibiotic exposure information associated with samples analyzed from total cohort of mother-infant dyads.

|  | Breastmilk (n = 85/91)^a^ | 1-month feces  (n =124/115)^a^ | 6-month feces  (n = 97/84)^a^ | *p* values |
| --- | --- | --- | --- | --- |
| Prenatal antibacterial exposure, % | 25/21 | 23/24 | 19/21 | 0.56/ 0.80 |
| Prenatal antifungal exposure, % | 6/5 | 5/6 | 4/6 | 0.87/ 0.98 |
| Perinatal antibacterial exposure, % | 29/28 | 26/27 | 21/23 | 0.49/ 0.69 |
| Postnatal antibacterial exposure^b^ - maternal, % | 14/20 | 23/22 | 13/16 | 0.17/ 0.58 |
| Postnatal antibacterial exposure^b^ - infant, % | 4/2 | 2/2 | 12/13 | 0.002/ <0.001 |
| Postnatal antifungal exposure^c^ –  infant, % | 6/4 | 6/9 | 6/8 | 0.99/ 0.52 |

^a^number of samples remaining after quality control procedures after sequencing (see Methods). Due to differences in sequencing output for bacteria vs fungi, the number of samples for their analyses differed as did the Table data for each kingdom (bacterial-associated data/fungal-associated data).

^b^postnatal antibiotic exposure for breastmilk and one-month feces is defined as occurring up until one month postpartum and not including perinatal (birth antibiotics), postnatal antibiotic exposure for six-month feces is defined as occurring between 1 and 6 months of age.

^c^ Information regarding maternal postnatal antifungal exposure was not available for this cohort.

Supplemental Table 4. Antibiotic exposure information associated with subset of samples included in network analyses.

|  | Breastmilk (n = 75)^a^ | 1-month feces (n = 100)^a^ | 6-month feces (n = 69)^a^ | *p* value |
| --- | --- | --- | --- | --- |
| Prenatal antibacterial exposure, % | 20 | 25 | 16 | 0.35 |
| Prenatal antifungal exposure, % | 7 | 5 | 6 | 0.90 |
| Perinatal antibacterial exposure, % | 26 | 27 | 21 | 0.66 |
| Postnatal antibacterial exposure^b^ - maternal, % | 20 | 24 | 16 | 0.44 |
| Postnatal antibacterial exposure^b^ - infant, % | 3 | 2 | 13 | 0.003 |
| Postnatal antifungal exposure^c^ - infant, % | 4 | 5 | 7 | 0.67 |

^a^number of samples remaining after quality control procedures after sequencing and after including only samples with paired bacterial and fungal sequences (see Methods).

^b^postnatal antibiotic exposure for breastmilk and one-month feces is defined as occurring up until 1 month postpartum and not including perinatal (birth antibiotics), postnatal antibiotic exposure for 6-month feces is defined as occurring between 1 and 6 months of age.

^c^No information available for maternal postnatal antifungal exposure

Supplemental Table 5. Clinical characteristics associated with samples grouped according to birth mode.

|  | Vaginal birth | C-section birth | *p* value |
| --- | --- | --- | --- |
| Number of samples  Breastmilk  1-month feces  6-month feces | 70/75  104/93  82/72 | 14/15  18/19  13/11 | NA^a^  NA  NA |
| Pre-pregnancy maternal BMI, mean kg/m^2^±SD  Breastmilk  1-month feces  6-month feces | 27.3±5.0/26.3±5.1  26.3±4.8/26.5±4.5  26.4±4.8/26.2±4.8 | 28.1±4.8/28.7±4.8  28.5±4.6/28.5±4.8  26.3±4.5/26.8±3.9 | 0.59/0.10  0.08/0.11  0.94/0.68 |
| Infant sex, % female  Breastmilk  1-month feces  6-month feces | 56/57  52/49  59/57 | 43/40  44/37  62/36 | 0.55/0.34  0.74/0.45  1/0.34 |
| Maternal age, mean yrs±SD  Breastmilk  1-month feces  6-month feces | 31.2±3.8/31.0±4.0  30.8±3.8/31.0± 3.9  31.2±4.2/31.1±4.0 | 33.8±3.7/33.5±3.8  32.7±3.9/32.6± 4.8  33.3±4.0/32.5±4.6 | 0.03/0.03  0.06/0.18  0.10/0.35 |
| Gestational age at birth, mean weeks±SD  Breastmilk  1-month feces  6-month feces | 39.7±1.1/39.7±1.1  39.8±1.1/39.8± 1.2  39.8±1.2/39.9±1.1 | 39.9±1.2/39.8±1.1  39.4±1.1/39.7± 1.0  39.6±1.3/39.8±1.1 | 0.62/0.79  0.16/0.64  0.68/0.79 |
| Infant race, % Group 1^a^  Breastmilk  1-month feces  6-month feces | 17/15  15/18  14/13 | 14/20  17/21  23/27 | 1/0.93  1/0.99  0.66/0.44 |
| Maternal race, % Group 1^b^  Breastmilk  1-month feces  6-month feces | 18/16  14/15  14/10 | 14/13  11/12  15/9 | 0.98/1  1/0.86  1/1 |
| Prenatal antibacterial exposure, %  Breastmilk  1-month feces  6-month feces | 29/24  25/26  20/21 | 7/7  17/21  8/9 | 0.18/0.25  0.64/0.88  0.52/0.62 |
| Prenatal antifungal exposure, %  Breastmilk  1-month feces  6-month feces | 6/5  5/7  5/7 | 7/7  6/5  0/0 | 1/1  1/1  0.94/0.82 |
| Perinatal antibacterial exposure, %  Breastmilk  1-month feces  6-month feces | 14/13  13/12  9/10 | 100/100  100/100  100/100 | <0.001/<0.001  <0.001/<0.001  <0.001/<0.001 |
| Postnatal antibacterial exposure^c^ – maternal, %  Breastmilk  1-month feces  6-month feces | 16/21  23/24  13/17 | 14/13  17/11  8/9 | 1/0.72  0.77/0.34  0.90/0.84 |
| Postnatal antibacterial exposure^c^ – infant, %  Breastmilk  1-month feces  6-month feces | 4/3  2/2  12/14 | 0/0  0/0  15/18 | 1/1  1/1  1/1 |
| Postnatal antifungal exposure^d^ – infant, %  Breastmilk  1-month feces  6-month feces | 7/5  7/6  6/7 | 0/0  6/0  8/9 | 0.68/0.82  1/0.48  1/1 |

^a^NA, not applicable

^b^ The only race category not included in Group 1 is White. Group 1 (vaginal birth) infant race composition (subject n for bacterial/fungal analysis) by sample type: Breastmilk; American Indian/Alaska native 2/2, Asian 1/2, Black or African American 6/4, More than one race 1/1, Other 2/2. 1-month feces; American Indian/Alaska native 3/3, Asian 2/3, Black or African American 5/5, More than one race 3/2, Other 2/3. 6-month feces; American Indian/Alaska native 2/2, Asian 0/1, Black or African American 5/3, More than one race 2/2, Other 2/1. Group 1 (C-section birth) infant race composition by sample type: Breastmilk; American Indian/Alaska native 0/0, Asian 1/1, Black or African American 0/1, More than one race 0/0, Other 1/1. 1-month feces; American Indian/Alaska native 0/0, Asian 0/1, Black or African American 2/1, More than one race 0/1, Other 1/1. 6-month feces; American Indian/Alaska native 0/0, Asian 1/1, Black or African American 0/0, More than one race 1/1, Other 1/1.

^c^ Group 1 (vaginal birth) maternal race composition by sample type: Breastmilk; American Indian/Alaska native 2/2, Asian 2/3, Black or African American 6/4, More than one race 1/1, Other 2/2. -1month feces; American Indian/Alaska native 3/3, Asian 3/3, Black or African American 4/4, More than one race 3/2, Other 1/2. 6-month feces; American Indian/Alaska native 2/2, Asian 1/1, Black or African American 4/3, More than one race 2/1, Other 2/0. Group 1 (C-section birth) maternal race composition by sample type: Breastmilk; American Indian/Alaskan native 0/0, Asian 0/0, Black or African American 0/1, More than one race 0/0, Other 2/1. 1-month feces; American Indian/Alaskan native 0/0, Asian 0/0, Black or African American 2/1, More than one race 0/0, Other 0/1. 6-month feces; American Indian/Alaskan native 0/0, Asian 0/0, Black or African American 0/0, More than one race 0/0, Other 2/1.

^c^postnatal antibiotic exposure for breastmilk and 1-month feces is defined as occurring up until 1 month postpartum and not including perinatal (birth antibiotics), postnatal antibiotic exposure for 6-month feces is defined as occurring between 1 and 6 months of age.

^d^Information regarding maternal postnatal antifungal exposure was not available for this cohort.

Supplemental Table 6. PCR cycle time (C_T_) determinations from breastmilk DNA isolated by different methods using primers targeting bacterial (16S) and fungal (ITS2) DNA.

|  | 16S | | | ITS2 | | |
| --- | --- | --- | --- | --- | --- | --- |
|  | PowerSoil | PowerFood | PowerSoil Pro | PowerSoil | PowerFood | PowerSoil Pro |
| 250 µl Breastmilk | 34.5 | 32.3 | 32.3 | 31.0 | 35.6 | 32.5 |
| 1 ml Breastmilk | 34.1 | 32.9 | 31.4 | 31.9 | 32.7 | 31.8 |
| Breastmilk pellet | 32.1 | 32.7 | 30.3^a^ | 32.1 | 31.8 | 30.0^a^ |

^a^PowerSoil Pro kit produced highest quantities of amplicon DNA for both bacteria and fungi.

Supplemental Table 7. Abundance and prevalence of bacterial taxa in breastmilk and infant feces.

| Top 10 abundant bacterial taxa | |  | Top 10 prevalent bacterial taxa | |
| --- | --- | --- | --- | --- |
| **Breastmilk (n = 85)** | **Sum of RAPs^1^** |  | **Breastmilk (n = 85)** | **# Samples** |
| *Streptococcus* | 1924 |  | *Streptococcus* | 85 |
| *Staphylococcus* | 1921 |  | *Staphylococcus* | 85 |
| *Sphingomonas* | 578 |  | *Cutibacterium* | 81 |
| *Corynebacterium* | 410 |  | *Corynebacterium* | 77 |
| *Brevundimonas* | 298 |  | *Bacillus* | 73 |
| *Cutibacterium* | 271 |  | *Unclassified Class Bacilli* | 73 |
| *Methylobacterium* | 227 |  | *Methylobacterium* | 70 |
| *Veillonella* | 221 |  | *Chryseobacterium* | 70 |
| *Gemella* | 209 |  | *Gemella* | 68 |
| *Mycobacterium* | 154 |  | *Veillonella* | 67 |
|  |  |  |  |  |
| **1 month feces (n = 124)** |  |  | **1 month feces (n = 124)** |  |
| *Bifidobacterium* | 2222 |  | *Bifidobacterium* | 124 |
| *Unclassified Family Enterobacteriaceae* | 2071 |  | *Unclassified Family Enterobacteriaceae* | 124 |
| *Bacteroides* | 1919 |  | *Bacteroides* | 124 |
| *Clostridium* | 1578 |  | *Clostridium* | 124 |
| *Veillonella* | 965 |  | *Streptococcus* | 124 |
| *Unclassified Order Enterobacterales* | 765 |  | *Veillonella* | 123 |
| *Streptococcus* | 586 |  | *Unclassified Order Enterobacterales* | 123 |
| *Haemophilus* | 266 |  | *Staphylococcus* | 121 |
| *Blautia* | 255 |  | *Lactobacillus* | 120 |
| *Erysipelatoclostridium* | 227 |  | *Haemophilus* | 116 |
|  |  |  |  |  |
| **6 month feces (n = 97)** |  |  | **6 month feces (n = 97)** |  |
| *Bifidobacterium* | 3041 |  | *Bifidobacterium* | 97 |
| *Veillonella* | 1288 |  | *Veillonella* | 97 |
| *Bacteroides* | 1266 |  | *Unclassified Family Enterobacteriaceae* | 97 |
| *Unclassified Family Enterobacteriaceae* | 1069 |  | *Streptococcus* | 97 |
| *Clostridium* | 518 |  | *Unclassified Order Enterobacterales* | 96 |
| *Streptococcus* | 330 |  | *Bacteroides* | 94 |
| *Blautia* | 247 |  | *Clostridium* | 94 |
| *Erysipelatoclostridium* | 158 |  | *Actinomyces* | 94 |
| *Enterococcus* | 157 |  | *Enterococcus* | 93 |
| *Akkermansia* | 150 |  | *Lactobacillus* | 90 |

^1^RAPs, relative abundance percentages, as described in Methods

Supplemental Table 8. Abundances and prevalences of fungal taxa in breastmilk and infant feces.

| Top 10 abundant fungal taxa | |  | Top 10 prevalent fungal taxa | |
| --- | --- | --- | --- | --- |
| **Breastmilk (n=91)** | **Sum of RAPs^1^** |  | **Breastmilk (n=91)** | **# Samples** |
| *Paecilomyces dactylethromorphus* | 2400 |  | *Sarocladium kiliense* | 59 |
| *Fusarium equiseti* | 1530 |  | *Paecilomyces dactylethromorphus* | 59 |
| *Malassezia restricta* | 851 |  | *Fusarium equiseti* | 59 |
| *Cladosporium ossifragi* | 638 |  | *Malassezia restricta* | 43 |
| *Vanrija pseudolonga* | 307 |  | *Candida albicans* | 38 |
| *Candida albicans* | 272 |  | *Sarocladium oryzae* | 33 |
| *Malassezia arunalokei* | 262 |  | *Cladosporium ossifragi* | 27 |
| *Nigrospora bambusae* | 262 |  | Thermoascaceae | 25 |
| *Fusarium fujikuroi* | 213 |  | Cladosporium | 23 |
| Aspergillus | 197 |  | Aspergillus | 23 |
|  |  |  |  |  |
| **1 month feces (n=115)** |  |  | **1 month feces (n=115)** |  |
| *Paecilomyces dactylethromorphus* | 1870 |  | *Paecilomyces dactylethromorphus* | 70 |
| *Candida albicans* | 1550 |  | *Sarocladium kiliense* | 66 |
| *Malassezia restricta* | 1350 |  | *Candida albicans* | 47 |
| *Fusarium equiseti* | 757 |  | *Malassezia restricta* | 42 |
| Aspergillus | 733 |  | *Fusarium equiseti* | 41 |
| *Cladosporium ossifragi* | 618 |  | Cladosporium | 27 |
| *Candida parapsilosis* | 495 |  | *Candida parapsilosis* | 26 |
| *Nigrospora bambusae* | 383 |  | Aspergillus | 26 |
| Didymellaceae | 381 |  | *Sarocladium oryzae* | 24 |
| *Malassezia arunalokei* | 344 |  | *Cladosporium ossifragi* | 22 |
|  |  |  |  |  |
| **6 month feces (n=84)** |  |  | **6 month feces (n=84)** |  |
| *Paecilomyces dactylethromorphus* | 1220 |  | Sarocladium kiliense | 51 |
| *Malassezia restricta* | 1070 |  | *Paecilomyces dactylethromorphus* | 50 |
| *Candida albicans* | 787 |  | *Malassezia restricta* | 44 |
| *Candida parapsilosis* | 607 |  | *Candida albicans* | 41 |
| *Cladosporium ossifragi* | 438 |  | *Fusarium equiseti* | 38 |
| *Fusarium equiseti* | 426 |  | *Cladosporium ossifragi* | 31 |
| Aspergillus | 383 |  | Aspergillus | 26 |
| *Malassezia arunalokei* | 336 |  | Cladosporium | 25 |
| Didymellaceae | 292 |  | *Candida parapsilosis* | 21 |
| *Vanrija pseudolonga* | 220 |  | Didymellaceae | 20 |

^1^RAPs, relative abundance percentages, as described in Methods

Supplemental Table 9. Statistical comparisons of microbial diversity features of bacterial (16S) and fungal (ITS2) communities by antibiotic exposure and delivery mode variable for each sample type.

| Comparison groups | Sample type | 16S alpha^2^  *p*-value | 16S beta^3^  *p-*value | ITS alpha^2^  *p*-value | ITS beta^3^  *p*-value |
| --- | --- | --- | --- | --- | --- |
| Prenatal antibacterial vs. none | Breastmilk | 0.49 | 0.14 | 0.2 | 0.13 |
|  | 1-month feces | 0.99 | 0.78 | 0.33 | 0.06 |
|  | 6-month feces | 0.64 | 0.94 | 0.6 | 0.89 |
| Prenatal antifungal vs. none | Breastmilk | 0.87 | 0.62 | 0.44 | 0.31 |
|  | 1-month feces | 0.39 | 0.23 | 0.9 | 0.55 |
|  | 6-month feces | 0.78 | 0.28 | 0.24 | 0.97 |
| Perinatal antibacterial vs. none | Breastmilk | 0.72 | 0.21 | 0.29 | 0.29 |
|  | 1-month feces | 0.85 | 0.061 | 0.7 | 0.22 |
|  | 6-month feces | 0.78 | **0.044** | 0.65 | 0.19 |
| Postnatal antibacterial-maternal vs none | Breastmilk | 0.32 | 0.78 | 0.25 | 0.87 |
|  | 1-month feces | **0.035** | 0.25 | 0.82 | 0.67 |
|  | 6-month feces | 0.85 | 0.51 | 0.79 | 0.83 |
| Postnatal antibacterial-infant vs. none | Breastmilk | 0.14 | **0.02**^1^ | 0.59 | 0.57 |
|  | 1-month feces | 0.68 | 0.13 | 0.19 | 0.88 |
|  | 6-month feces | 0.45 | 0.39 | **0.039** | 0.26 |
| Postnatal antifungal-infant vs. none | Breastmilk | 0.096 | 0.89 | 0.94 | 0.94 |
|  | 1-month feces | 0.45 | 0.8 | 0.6 | 0.92 |
|  | 6-month feces | 0.34 | 0.36 | 0.38 | 0.4 |
| Cesarean section vs vaginal birth | Breastmilk | 0.61 | 0.95 | 0.36 | 0.43 |
|  | 1-month feces | 0.90 | **0.004** | 0.72 | **0.034** |
|  | 6-month feces | 0.006 | **0.019** | 0.12 | 0.17 |

*p*-values <0.05 highlighted **bold**

^1^only 2 infants had postnatal antibacterial antibiotic exposure between birth and 1 month of age

^2^alpha diversity tested with linear mixed effect models

^3^beta diversity tested with PERMANOVA

Supplemental Table 10. Interkingdom connectedness values for birth mode and antibiotic exposure subgroups by sample type

| Analysis group | Sample type | Connectedness |
| --- | --- | --- |
| Vaginal / Cesarean birth | Breastmilk | 0.80 / 1.00 |
|  | 1-month feces | 0.67 / 0.70 |
|  | 6-month feces | 0.78 / 0.91 |
| Perinatal antibiotics Y/N | Breastmilk | 1.00 / 0.69 |
|  | 1-month feces | 0.73 / 0.69 |
|  | 6-month feces | 0.83 / 1.05 |
| Postnatal antibacterial-maternal Y/N | Breastmilk | 0.78 / 0.60 |
|  | 1-month feces | 0.89 / 0.63 |
|  | 6-month feces | 0.89 / 0.74 |
| Postnatal antibacterial-infant Y/N | Breastmilk | n/a^1^ / 0.82 |
|  | 1-month feces | n/a^1^ / 0.67 |
|  | 6-month feces | 0.82 / 0.83 |

^1^n/a, not analyzed because only 2 of 100 infants received antibiotics between birth and 1 month of age.
